# Temporal and spatial pattern of endophytic fungi diversity of *Camellia sinensis* (cv. Shu Cha Zao)

**DOI:** 10.1186/s12866-020-01941-1

**Published:** 2020-08-28

**Authors:** Zhenzhen Wu, Qingqing Su, Yuchen Cui, Hongzhu He, Jiali Wang, Yong Zhang, Yu Zhao, Hassan Abul, Yunqiu Yang, Yanhua Long

**Affiliations:** 1 School of Life Sciences, Anhui Agricultural University, Hefei, 230036 Anhui China; 2 State Key Laboratory of Tea Plant Biology and Utilization, Anhui Agricultural University, Hefei, 230036 Anhui China

**Keywords:** Shu Cha Zao, Endophytic fungi, Spatial and dynamic distribution, High-throughput sequencing, Tea

## Abstract

**Background:**

The experimental materials were a 60-year-old tea tree (*Camellia sinensis* cv. Shu Cha Zao; SCZ) (the mother plant) and 1-year-old and 20-year-old plants of SCZ that originated as mother plant cuttings. The aim of this study was to use high-throughput sequencing to study the spatial and dynamic distribution of endophytic fungi in different leaf niches (upper leaves, middle leaves, lower leaves) and rhizosphere soil on tea plants of different ages in the same garden.

**Results:**

Ascomycota (83.77%), Basidiomycota (11.71%), and Zygomycota (3.45%) were the dominant fungal phyla in all samples. *Cladosporium* (12.73%), *Zymoseptoria* (9.18%), and *Strelitziana* (13.11%) were the dominant genera in the leaf. Alpha diversity analysis revealed that endophytic communities in leaves differed from those in rhizosphere soil and different leaf niches had similar fungal diversity. Shannon’s indices and NMDS analysis indicated significant differences in fungal diversity and composition among the SCZ trees of different ages (*p* ≤ 0.01). The abundance of *Cladosporium* and *Zymoseptoria* decreased with increasing SCZ age, whereas the abundance of *Strelitziana* increased.

**Conclusions:**

The results illustrate variation in endophytic fungi among different niches on tea plants of different ages. The distribution of endophytic fungi in leaves of *C. sinensis* shows spatiotemporal variation.

## Background

Endophytic fungi are important microbial plant symbionts [1]. They live in healthy plant tissues either at certain growth stages or throughout their life history. Endophytic fungi inhabit many plant tissues, such as leaves, stems, bark, petioles, roots, and reproductive structures. These fungi cause no apparent disease symptoms, and they include latent pathogens and mycorrhizal fungi [1]. A notable endophytic fungus is *Taxomyces andreanae* from Pacific yew (*Taxus brevifolia*), which produces taxol and related anti-cancer substances [2]. Endophytic fungi have been isolated from moss [3], ferns [4], grasses [5], shrubs [6], conifers [7], and deciduous trees [8]. Most research has focused on their isolation, identification, diversity, metabolites, and host interactions [9–11].

*Camellia sinensis* is an evergreen shrub in the *Theaceae* family [12, 13]. In China, tea is made from the young leaves of *C. sinensis*, whereas the mature leaves are seldom used. The taste and health benefits of tea are related to the levels of polyphenols, caffeine, anthocyanins, and other ingredients in the leaves [14]. There is little information on the composition and distribution of endophytic fungi, as well as the active ingredients, from tea leaves in different plant parts or from trees of different ages.

Knowledge of the endophytic fungi of *C. sinensis* is mainly limited to diversity and distribution [15–17], how fungal distribution is affected by season, habitat [18], and leaf age [19]. Relatively few fungi have been isolated using traditional culture methods. Sequence analysis on 18S rDNA [20–22] and internal transcribed spacer (ITS) regions [23–25] is widely used in classification and identification of fungi. Further, high-throughput sequencing of amplicons has been used in studies on fungal community diversity [26, 27]. This method overcomes the problems that some endophytic fungi cannot grow on artificial media or are outcompeted by faster growing species. High-throughput sequencing of amplicons can provide sequence information of endophytes and allow comprehensive analysis.

Some studies on the relationship between a plant endophyte and its developmental stage or different vegetative organs using amplicon high-throughput sequencing have been published. Cregger used *Populus* as a model plant ecosystem and found that the fungal microbiome varied among leaves, stems, roots, and soils regardless of the plant genotype, and differed significantly between stems and soils [28]. In sugarcane, nearly half of the fungal OTUs inhabited the endophytic and exophytic compartments of roots, shoots, and leaves [29]. These communities originated from the native soil surrounding the plants, and plant organs were colonized via different patterns. Meanwhile, the dynamics of endophytic fungal communities were significantly influenced by plant genotype and plant growth stage in sugar beets. Endophytic fungal diversity during seedling growth and rosette formation were much lower than the diversity found during sucrose accumulation and tuber growth [30].

Endophytic fungi diversity, including changes during *C. sinensis* growth and development, has not been adequately studied. We used high-throughput sequencing technology to study the fungal community structure and diversity in the upper leaves, middle leaves, lower leaves, and rhizosphere soil of tea samples, to determine the changes related to different leaf niches or ages of the tea plants.

## Results

### OTU clustering and species annotation

The raw sequence data of all samples consisted of 5,060,529 reads prior to quality checking and assigning the reads to their respective samples. The average read length (± standard deviation) of reads before processing was 243.77 ± 11.20 bp. After quality trimming and assigning reads to different samples, there were 4,489,368 high-quality reads in the data set, with an average length (± standard deviation) of 246.45 ± 15.36 bp. A total of 3753 OTUs were generated after clustering at a 97% similarity level. Representative sequences for each OTU were screened for further annotation. Seven phyla, 33 classes, 117 orders, 271 families, 480 genera, and 762 species were identified from these sequences.

### Composition of fungal communities

**Fig. 1:**
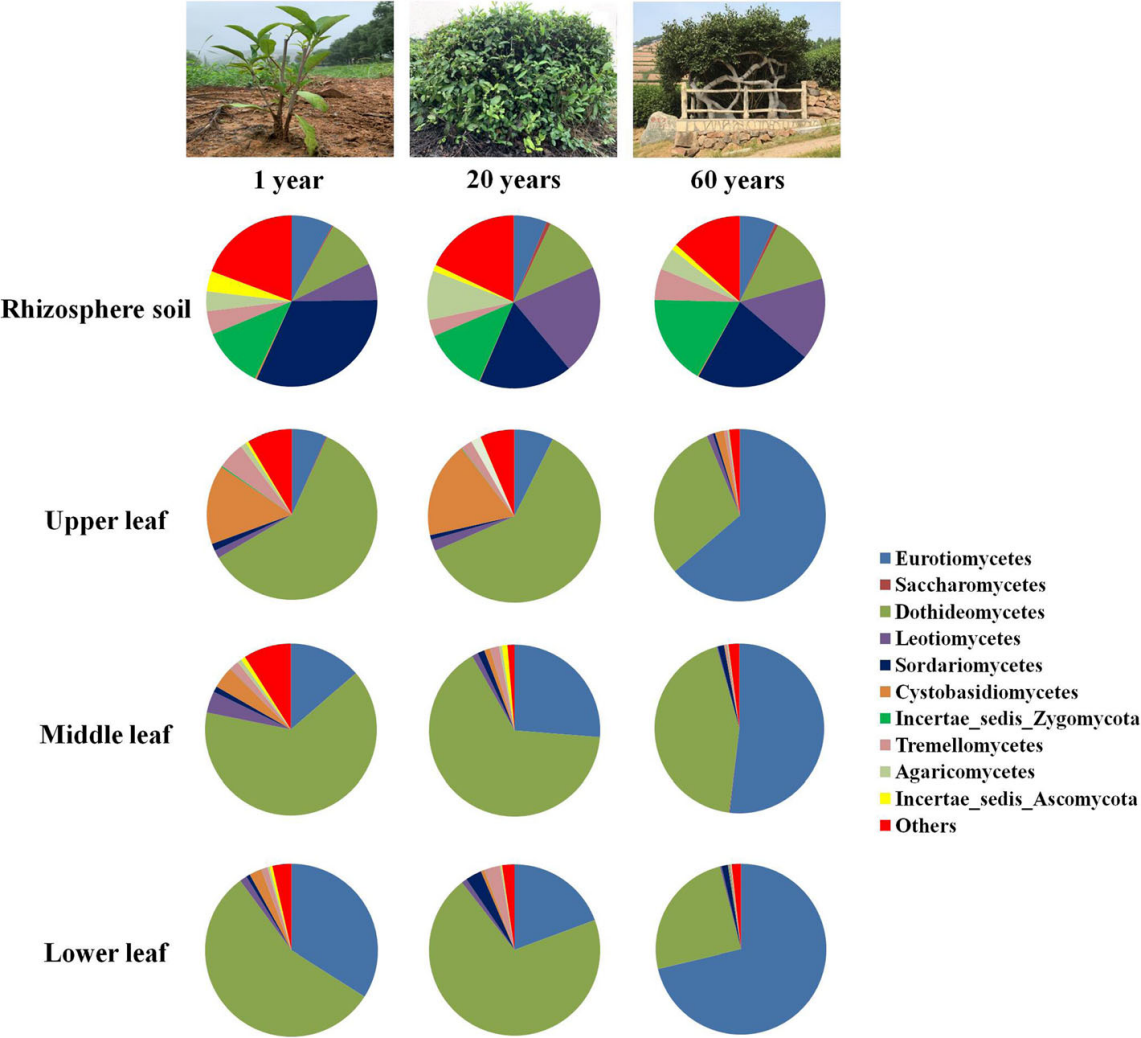
Relative abundance of dominant (>0.1%) fungal classes in leaf niches (upper leaf, middle leaf, lower leaf) and rhizosphere soil from plants of different ages

Ascomycota (83.77%), Basidiomycota (11.71%), and Zygomycota (3.45%) were the dominant phyla (Fig. S1). Dothideomycetes (52.8%) and Eurotiomycetes (32.67%) had a higher proportion in leaf samples of all plant ages compared to rhizosphere soil (11.41 and 6.95%, respectively). Sordariomycetes and Leotiomycetes were more abundant in rhizosphere soil (23.76 and 14.40%, respectively) than in leaf niches (1.25 and 1.45%, respectively) (Fig. 1). Cystobasidiomycetes and Microboiryomyceres were more abundant in the upper leaves than other leaf niches. Capnodiales (25.35%) and Chaetothyriales (13.93%) were the dominant orders in leaf niches (Fig. S1), while Mortierellales (12.10%), Hypocreales (11.47%) and Helotiales (8.53%) were the dominant orders in rhizosphere soil. The dominant families in the leaf niches were Davidiellaceae (14.65%), Incertae_sedis_Chaetothyriales (13.31%), Incertae_sedis_Dothideomycetes (12.71%) and Mycosphaerellaceae (9.86%). But Mortierellaceae (11.21%), Nectriaceae (4.86%), and Amphisphaeriaceae (4.27%) show a high proportion in the rhizosphere soil (Fig. S1). *Cladosporium* (12.73%), *Zymoseptoria* (9.18%), and *Strelitziana* (13.11%) were the dominant genera in the leaves (Fig. S1).

**Fig. 2:**
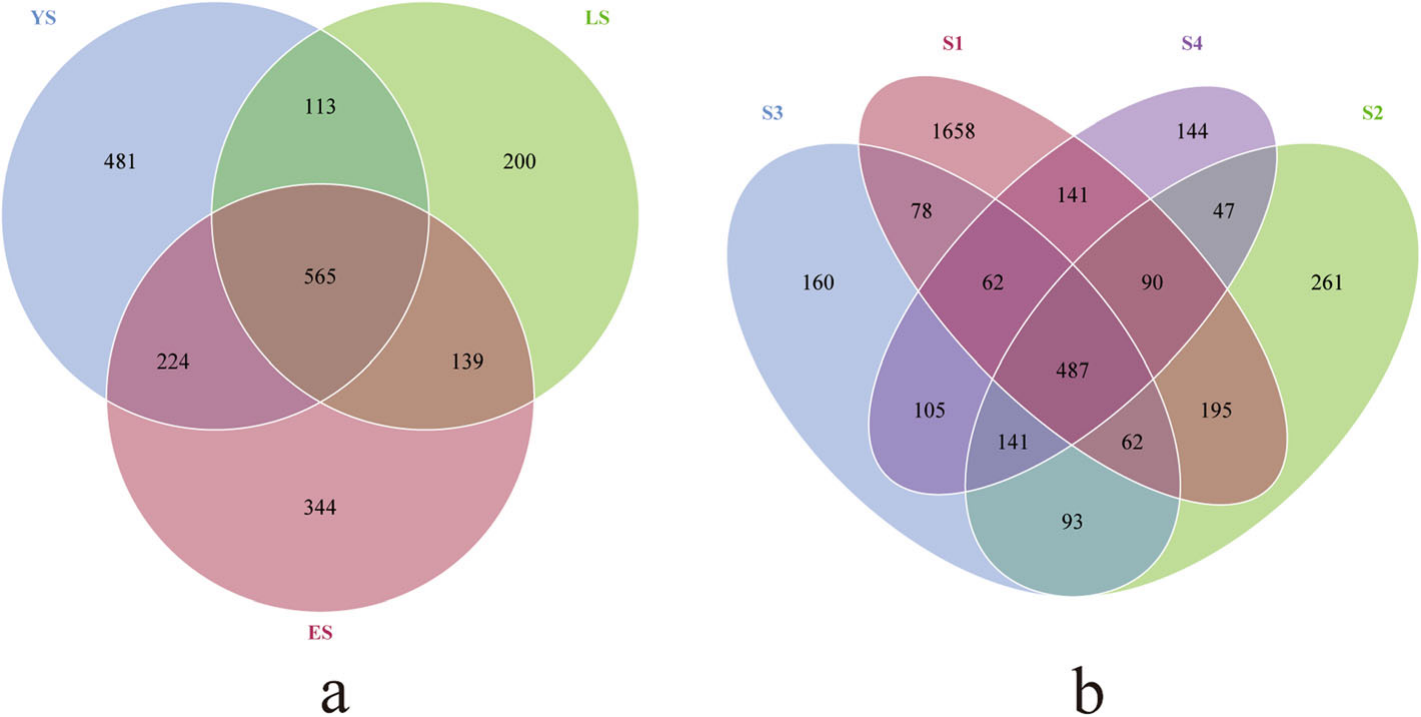
Venn diagrams showing number of shared OTUs among sample groups. **a** Number of shared OTUs among tea plants of different ages. **b** Number of shared OTUs among different niches (S1, rhizosphere soil; S2, upper leaf; S3, middle leaf; S4, lower leaf)

**Fig. 3:**
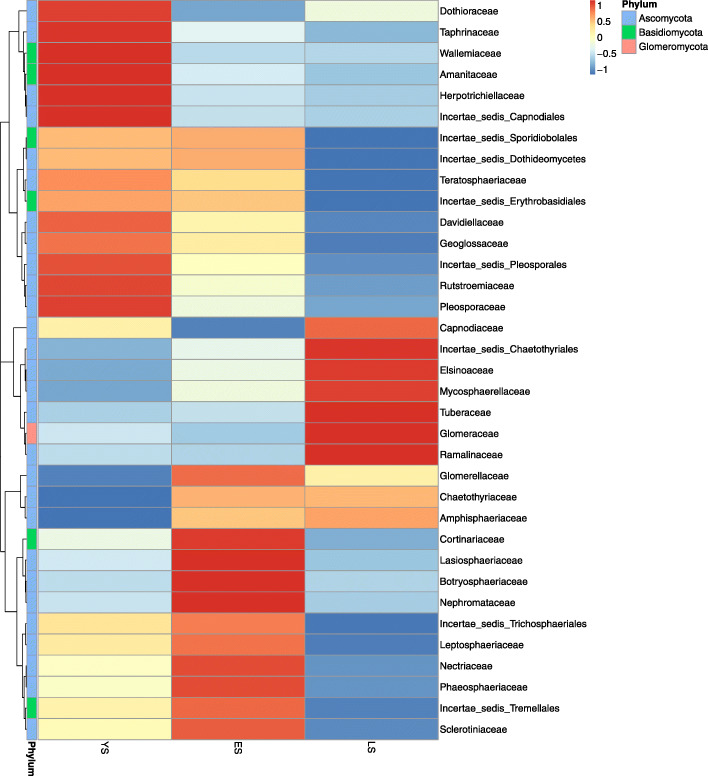
Heat maps of leaf endophytic fungal families in tea trees of different ages. Square colors shifted from dark blue toward red indicate higher abundance. (YS, 1-year-old tea plant; ES, 20-year-old tea plant; LS, 60-year-old mother plant)

A Venn diagram was constructed to highlight the similarities and differences in communities among different ages of plants and leaf/soil niches. The communities in YS, ES, and LS had 565 OTUs in common; the upper leaves, middle leaves, lower leaves, and rhizosphere soil had 487 OTUs in common (Fig. 2). Some OTUs appearing in the leaf endophytic fungi community were also detected in rhizosphere soil, which suggests the possibility of soil fungi colonizing leaves. The large number of common OTUs among samples from different-aged trees indicates that colonization patterns may be conserved during long-term evolution. The leaf endophytic fungal communities differed between LS and YS/ES (Fig. 3). We found that the relative abundance of some families among the top 35 OTUs, such as Dothioraceae, Taphrinaceae, Wallemiaceae, Amanitaceae, Herpotrichiellaceae, Incertae_sedis_Capnodiales, Incertae_sedis_Sporidiobolales, Incertae_sedis_Dothideomycetes, Davidiellaceae, Teratosphaeriaceae, Incertae_sedis_Erythrobasidiales, Geoglossaceae, Incertae_sedis_Pleosporales, Rutstroemiaceae, and Pleosporaceae, decreased with the increasing age of the plant. In contrast, the abundance of Incertae_sedis_Chaetothyriales, Elsinoaceae, Mycosphaerellaceae, Tuberaceae, Glomeraceae, and Ramalinaceae increased with tree age, and gradually became dominant in LS.

### Alpha rarefaction curves and alpha diversity

**Fig. 4:**
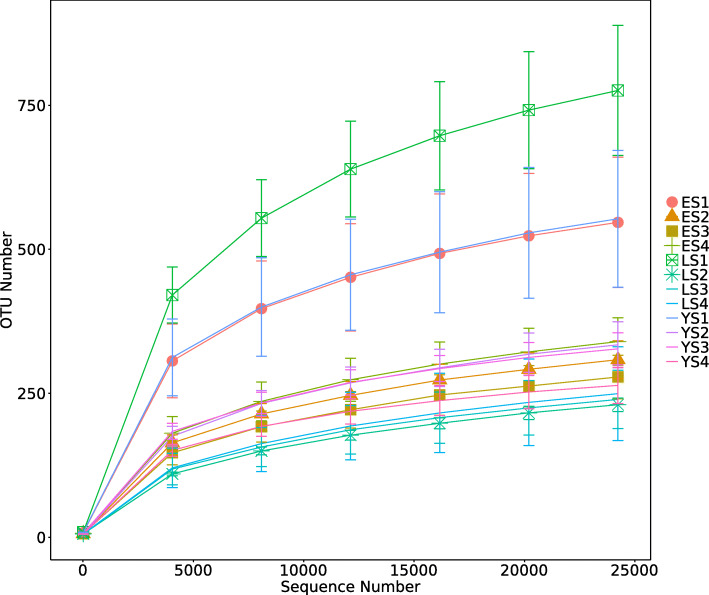
Rarefaction curves of OTUs in different samples (YS, 1-year-old tea plant; ES, 20-year-old tea plant; LS, 60-year-old tea plant; 1, rhizosphere soil; 2, upper leaf; 3, middle leaf; 4, lower leaf)

The rarefaction curves approached the plateau phase, indicating that it is unlikely that more fungal taxa would be detected with additional sequencing (Fig. 4). These curves showed the endophytic fungi communities were less diverse in leaves than in the rhizosphere soil, as evidenced by differences in number of OTUs between these communities.

**Fig. 5:**
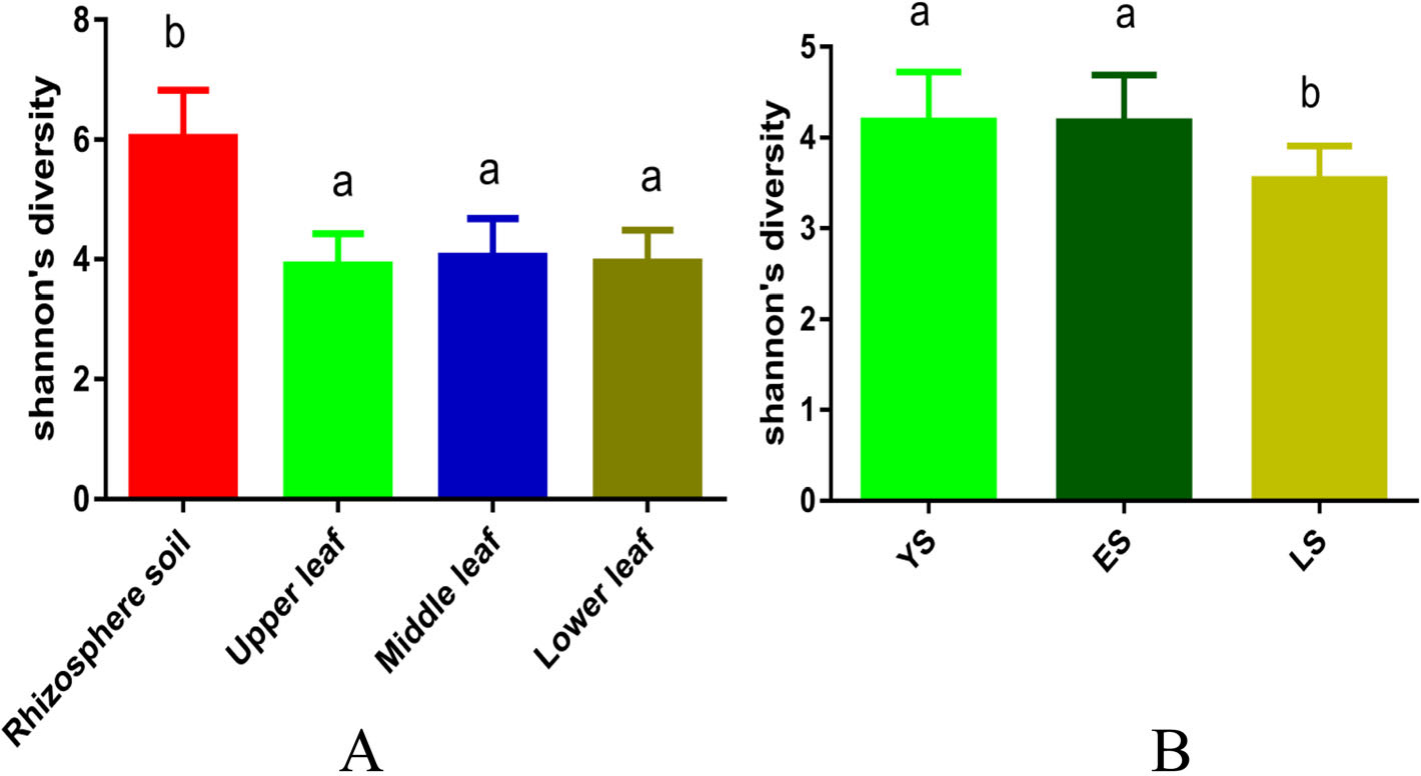
**A** Shannon’s diversity values for fungal communities across samples in categories of niche (rhizosphere soil, upper leaf, middle leaf, lower leaf); **B** Shannon’s diversity values for fungal communities across samples in different plant ages. Different letters above bars within plots represent significant differences (pairwise t tests, *p*≤0.01). Bars represent means ± SEM (*n* = 5). YS, 1-year-old tea plant; ES, 20-year-old tea plant; LS, 60-year-old mother plant

Community richness and diversity were analyzed using five alpha diversity indices: Chao1, Shannon’s, Simpson’s, ACE, and Goods_coverage (Table S1). The depth index (Goods_coverage) of each sample library was over 99% (99.2–99.8%), indicating that the sampling was reasonable. The Chao1 and ACE indices are indicative of fungal community richness, and Shannon’s and Simpson’s indices are indicative of fungal community diversity. Fungal richness and diversity were significantly higher in rhizosphere soil than in leaf samples (*p* < 0.0001) (Fig. 5a). The richness and diversity of fungi in the rhizosphere soil of the mother plant were highest among all samples (Table S1). The different leaf niches had similar fungal alpha diversity (Fig. 5a). The Shannon’s indices indicated that fungal diversity was greater in younger plants (YS and ES) than in the mother plant (LS) (*p* ≤ 0.01) but did not differ significantly between YS and ES (Fig. 5b).

### Beta diversity

**Fig. 6:**
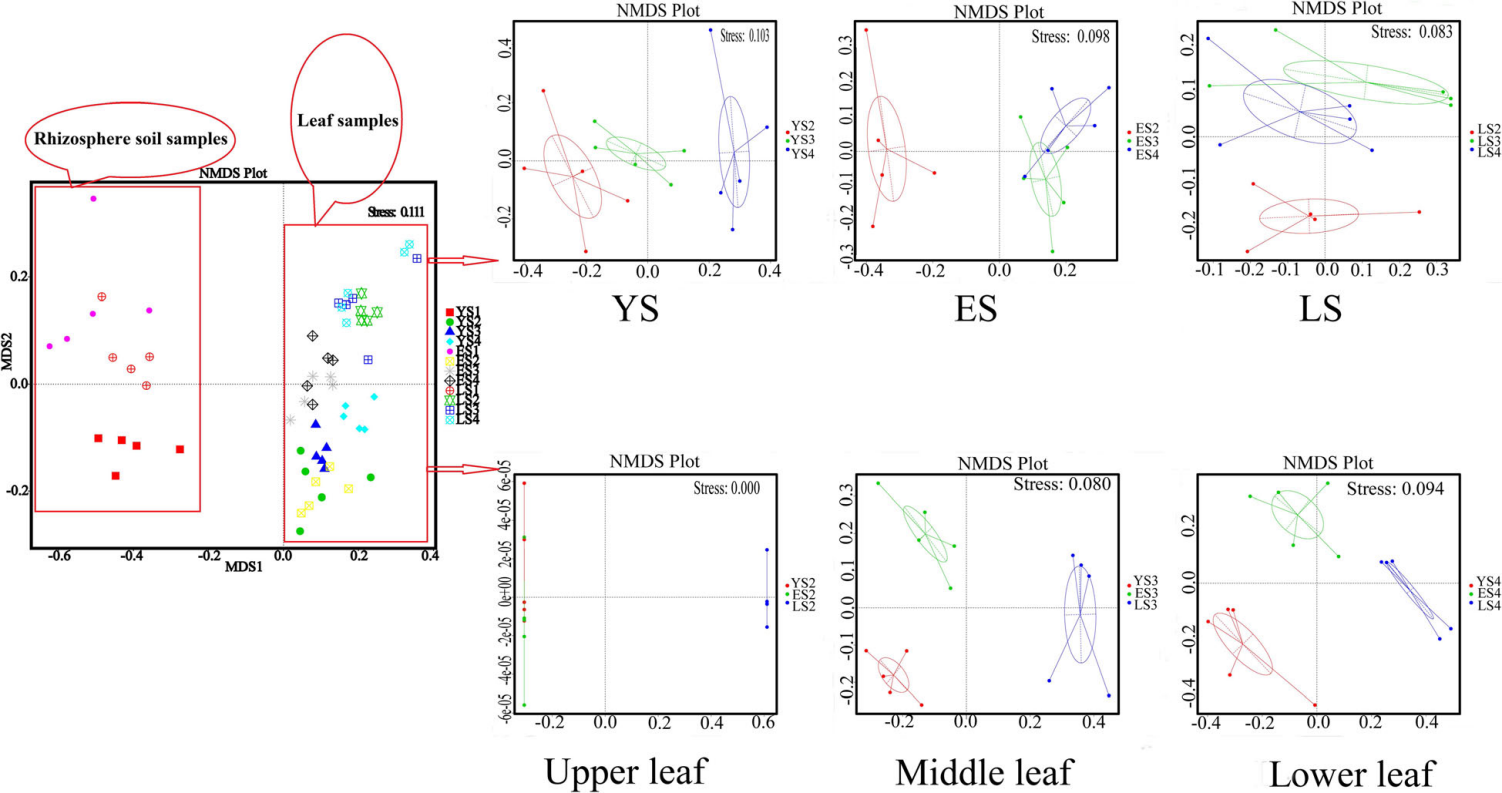
NMDS ordination of soil (1: rhizosphere soil) and leaf niches (2: upper leaf, 3: middle leaf, 4: lower leaf) in plants of different ages (YS, 1-year-old tea plant; ES, 20-year-old tea plant; LS, 60-year-old mother plant)

In NMDS analysis, the degree of difference between groups or in-group can be reflected through a multidimensional space. NMDS analysis revealed that the mycobiomes between rhizosphere soil samples and leaf samples were significantly distinguished (*R*^2^ = 0.23, *p <* 0.001) (Fig. 6, left).

On an analysis of the mycobiome composition of all samples, in the different leaf niches and the different tree ages, the samples were clearly separated (*R*^2^ = 0.022, *p <* 0.001) (Fig. 6, right). Independent analysis of YS, ES, and LS revealed significant differences respectively in the composition of endophytic fungi in the upper, middle, and lower niches (*p <* 0.001). Among them, the composition of endophytic fungi in the upper leaves of YS was not significantly different from that in the middle (*R*^2^ = 0.178, *p* = 0.092), but the differences between the upper and lower layers and the middle and lower layers were significant (*R*^2^ = 0.394, *p* = 0.007; *R*^2^ = 0.274, *p* = 0.006). In the comparative analysis of the endophytic fungi composition of the three leaf niches of ES, the pairwise differences between them were found to be significant (*p* < 0.05). Growth time affected the distribution of endophytic fungi. However, in the LS sample, the differences between the upper, middle, and lower layers were not significant. A horizontal comparison of tree age based on different leaf niches showed a significant difference between different ages of samples in middle and lower leaf layers (*p* < 0.001). However, in the upper leaf niche, the difference between YS and ES was not significant (*p* = 0.341). This could also be explained by the number of shared OTUs among YS and ES (789 OTUs), which was higher than that of YS and LS (678 OTUs) or ES and LS (704 OTUs) (Fig. 2).

## Discussion

### Richness, composition, and distribution of dominant endophytic fungi

Previous studies on endophytic fungi of tea plants have been conducted. However, there are shortcomings in the analyses of the population diversity of endophytic fungi. Traditional isolation and identification methods cannot detect the whole microbiome because it is difficult or impossible to cultivate many taxa. We analyzed the species richness, composition, and distribution of SCZ endophytic fungi in different leaf niches of different tree ages using high-throughput sequencing data, and compared them with rhizosphere soil fungal communities.

Ascomycota, Basidiomycota, and Zygomycota were the three dominant fungal phyla in SCZ, and these were also reported as the dominant phyla in sugar beets [14]. Dothideomycetes, Eurotiomycetes, Sordariomycetes, and Leotiomycetes were the dominant classes which was consistent with results on vascular plants from the high arctic zone [4]. In tropical and temperate plants, the major class of endophytes was reported to be Sordariomycetes, followed by Dothideomycetes and Leotiomycetes [31–33]. It appears that the dominant endophytic fungi are consistent in different plant species.

*Colletotrichum* sp., *Pestalotiopsis* sp., *Guignardia* spp., *Phomopsis* sp., *Macrophoma* sp., *Aspergillus* sp., *Candida* sp., *Thamidium* sp., *Alterinaria* sp., and *Fusarium* spp. were reported as dominant genera in tea trees [34–36]. However, *Cladosporium*, *Strelitziana*, *Zymoseptoria*, *Pseudeurotium*, *Pseudoramichloridium*, *Penicillifer*, *Trichoderma*, *Paraconiothyrium*, *Melanconiella*, and *Saccharomycopsis* showed high richness in our study based on the sequencing data. The different results between our study and previous studies may be related to the limitations of traditional separation and culture methods, different geographical distance, environmental conditions, and differences among the tea varieties.

**Fig. 7:**
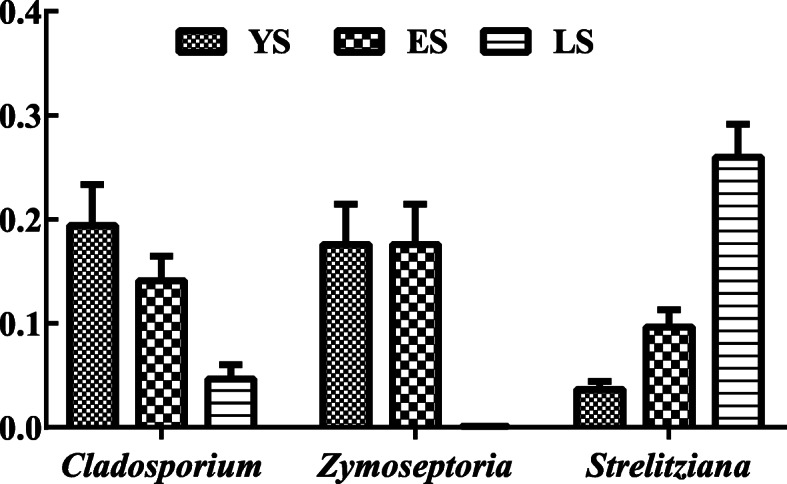
Competition map among* Cladosporium*, *Zymoseptoria*, and *Strelitziana*. (YS, 1-year-old tea plant; ES, 20-year-old tea plant; LS, 60-year-old mother plant)

During coexistence and evolution, some endophytic fungi have evolved to be integral partners of plants [34]. *C. sinensis* and its endophytic fungi may have formed stable relationships during their coevolution [18]. Many fungal species colonizing within the SCZ leaves have formed a dynamic equilibrium through continuous interspecific competition while under the influence of environmental factors. In SCZ, the levels of *Cladosporium* and *Zymoseptoria* decreased with increasing SCZ age, while the level of *Strelitziana* increased (Fig. 7). This suggests competition among *Cladosporium, Zymoseptoria,* and *Strelitziana* after their colonization.

The OTU abundance between different leaf niches and rhizosphere soil, showed significant differences (Fig. 2). The richness of microbial composition in rhizosphere soil is much higher than that in the roots or other plant organs. Microorganisms in the soil can gradually colonize the plant through the roots. We found that Sordariomycetes and Leotiomycetes were more abundant in rhizosphere soil than in leaf niches. Sordariomycetes were found to be soil decomposers in other studies [36, 37] and this could explain their abundance in the rhizosphere.

### Characteristics of temporal and spatial distribution

Pairwise differences between the three leaf niches of ES were significant. However, there was no significant difference of LS in the pairwise analysis. There were a considerable number of OTUs in the leaves of younger plants (YS and ES) than the mother plant (LS) (Fig. 3). The diversity difference of fungal communities between YS and ES was not significant, but it was significant among YS, ES, and the mother plant (Figs. 5 and 6). The degree of difference in endophytic fungal diversity is proportional to the difference in plant age. As the plant grew for 20 years, the colonization of leaf endophytic fungi at different heights formed a significant difference, but the 20-year growth period was insufficient for the endophytic fungi to evenly distribute throughout the three leaf niches. These analyses showed a pattern in the distribution of endophytic fungi in different leaf niches of the same plant. At the same time, the composition and distribution of endophytic fungi in plants showed differences associated with the plant age. The distribution of endophytic fungi in leaves of *C. sinensis* reflected spatiotemporal variation. *C. sinensis* has a long growing period and showed relatively low species diversity of endophytic fungi in this study. Changes of the major chemical components of leaves, such as tea polyphenols (catechins, gallic acid, anthocyanidin) and alkaloids (caffeine, theophylline, theobiomine) may affect the colonization of endophytic fungi. During colonization, only the endophytic fungi that tolerated these special chemical components could continue to survive. This would make the species composition and richness of endophytic fungi in leaves of ancient trees relatively low.

Fungal endophytes can be transmitted, horizontally or vertically, in the healthy foliage of woody plants [38]. Although horizontal transmission is considered to be the main transmission approach, some fungi are transmitted vertically. In this study, some OTUs shared among the leaf niches were also present in the rhizosphere soil. This may be caused by the vertical transmission and colonization of rhizosphere soil fungi (Fig. 2). Abundant shared OTUs existed in all samples with different ages (Fig. 2). Parts of the endophytic fungal communities of 1-year-old and 20-year-old SCZ plants may have been inherited from the mother plant.

In this study, the YS and ES plants were derived from cuttings of one mother plant, which was the only ancient tea plant in the garden. This sampling strategy avoided seasonal, genetic, and geographical variations among the treatments. Therefore, we were able to assess the mycobiome diversity solely in relation to different leaf niches from different aged SCZ plants.

## Conclusion

Our analyses showed an order in the distribution of endophytic fungi in different leaf niches of the same plant. However, the composition and distribution of endophytic fungi in the plants varied with their age. *Cladosporium*, *Zymoseptoria*, and *Strelitziana* were the dominant genera in leaves. The abundance of *Cladosporium* and *Zymoseptoria* decreased with increasing SCZ age, while the abundance of *Strelitziana* increased. The distribution of endophytic fungi in SCZ leaves exhibited spatiotemporal variation.

The SCZ fungal community significantly differed across the soil–upper leaf–middle leaf–lower leaf landscape and among trees of different ages. These findings provide information about the composition and diversity of endophytic fungi communities in tea plants of different ages, and will be useful in further research on the co-evolution and adaptation of endophytic fungi and tea trees. The data also provide a reference for research on endophytic microbes in other plant species.

## Methods

### Sample collection

**Fig. 8:**
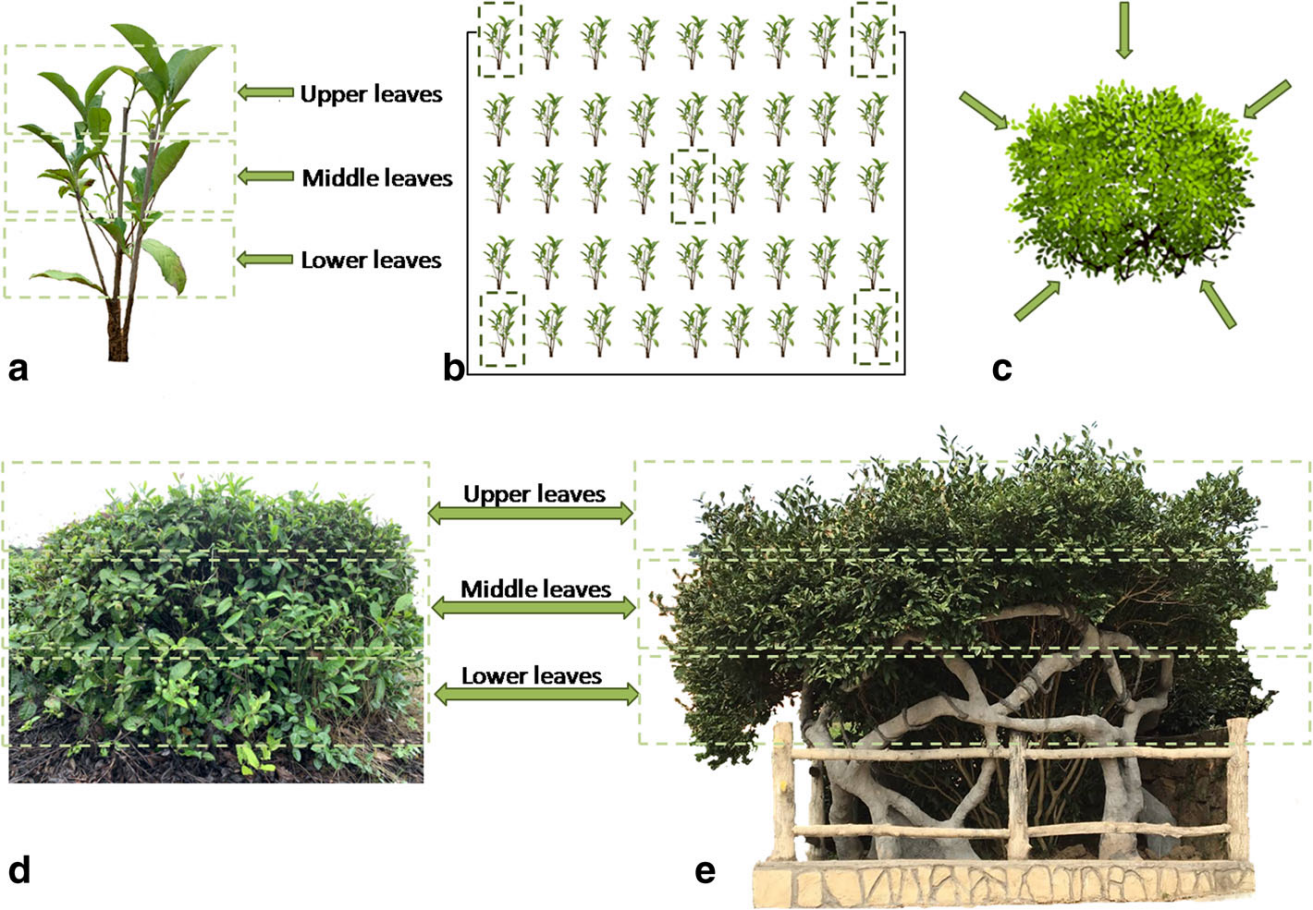
Sampling strategy diagram. (**a**, **d**, **e**: the sampling of upper leaves, middle leaves, and lower leaves from YS, ES, and LS; **b**: five plants of YS were randomly selected in the field; **c**: Top view of canopy, five directions were chosen on average around a tree)

To avoid the sampling variation caused by location differences, the variety Shu Cha Zao (SCZ) of *Camellia sinensis* located in the 916 tea plantation (Shucheng county, Lu’an, China) was selected as the main object. In this tea garden, there is one tea tree (regarded as the mother plant) over 60 years old, and this tree was named as 60-year-old SCZ (LS). Cuttings, originating from the mother plant, were cultivated from 1997 until 2017 and were named as 20-year-old SCZ (ES). One-year-old SCZ (YS) plants were cuttings from the mother plant taken in 2016. The upper leaves, middle leaves, lower leaves, and rhizosphere soil (Table 1) were collected from YS, ES, and LS in 2017. The sampling strategy is shown in Fig. 8. For YS, five plants were randomly selected in the same field (Fig. 8b). Based on height, the upper leaves, middle leaves, and lower leaves were collected respectively (Fig. 8a, d, e). Five ES plants were also randomly selected. Each ES was divided into five equal parts from top view, and leaves were collected from proximal, median and distal parts (the upper leaves, middle leaves, lower leaves) of the same branch in each region (Fig. 8c). Leaves from the same niche of one ES plant were mixed. For the one 60-year-old plant (LS, mother plant), samples were collected using the same method as described for ES. Leaves from each region were regarded as replications. The rhizosphere soil was sampled at a depth of 15–20 cm and about 6 mm away from the rhizoplane of each plant with five repetitions. All of the samples were placed on dry ice, immediately, transported to laboratory, and stored at − 80 °C for further experiments.

**Table 1:** Names (coordinates), parts, and serial numbers of materials

Sample name (coordinates)	Material position	Number
1-year-old Shu Cha Zao/YS (31°19′7″N, 117°1′25″E)	Rhizosphere soil	YS1
	Upper leaf	YS2
	Middle leaf	YS3
	Lower leaf	YS4
20-year-old Shu Cha Zao/ES (31°19′13″N, 117°1′21″E)	Rhizosphere soil	ES1
	Upper leaf	ES2
	Middle leaf	ES3
	Lower leaf	ES4
60-year-old Shu Cha Zao/LS (31°19′38″N, 117°1′57″E)	Rhizosphere soil	LS1
	Upper leaf	LS2
	Middle leaf	LS3
	Lower leaf	LS4

### DNA extraction and PCR amplification

To isolate microorganisms, all of the leaf samples were washed with tap water, soaked in 75% ethanol for 5 min, rinsed three times with sterile water, surface-sterilized for 5 min in 1% sodium hypochlorite, washed three times with sterile water, and then dried with sterile filter paper. The samples were ground into powder in liquid nitrogen using a mortar and pestle. Powder was transferred into a 50 mL tube containing 10 mL sterile water and centrifuged at 200×*g* for 20 min, 500×*g* for 20 min and 16,500×*g* for 15 min at 4 °C. Total genomic DNA was extracted from the precipitate using the CTAB method with slight modifications [39].

Polymerase chain reaction (PCR) was performed to amplify the ITS1 regions of the fungal ITS rRNA genes using primers ITS5-1737F (5′-GGA AGT AAA AGT CGT AAC AAG G-3′) and ITS2-2043R (5′-GCT GCG TTC TTC ATC GAT GC-3′). Each 30 μL PCR reaction mixture contained 15 μL Phusion Master Mix (2×, GenStar), 3 μL Primer (2 μM), 10 ng DNA, and 2 μL ddH_2_O. All of the samples were replicated three times. The PCR was performed using GeneAmp PCR System 9700 with the following standard procedure: initial denaturation at 98 °C for 1 min, followed by 30 cycles of 98 °C for 10 s, annealing at 50 °C for 30 s, 72 °C for 30 s, and final extension at 72 °C for 5 min. Then, the PCR products were analyzed by electrophoresis on a 2% (v/v) agarose gel (100 V, 40 min). The gel was stained with ethidium bromide, and bands were photographed on an ultraviolet light transilluminator.

### Library preparation and sequencing

cDNA libraries were generated using the TruSeq®DNA PCR-Free Sample Preparation Kit (Illumina, San Diego, CA, USA) with index codes following manufacturer recommendations. The library quality was assessed on the Qubit@2.0 Fluorometer (Thermo Scientific, Waltham, MA, USA) and Agilent Bioanalyzer 2100 system (Agilent, Palo Alto, CA, USA). The library was sequenced on the Illumina HiSeq 2500 platform and 250 bp paired-end reads were generated at Novogene (Beijing, China) [40].

### Statistical analysis

Sequences were analyzed using Uparse software (v7.0.1001, http://drive5.com/uparse/) [41]. Sequences with ≥97% similarity were assigned to the same OTU. A representative sequence for each OTU was screened for further annotation. Each representative sequence was annotated with taxonomic information from the Unite Database (https://unite.ut.ee/) [42] based on BLAST algorithm, which was calculated using QIIME (Version 1.9.1) (http://qiime.org/scripts/assign_taxonomy.html). To obtain the phylogenetic relationships among different species and differences in dominant species among different samples (groups), multiple sequence alignments were conducted using MUSCLE software (Version 3.8.31, http://www.drive5.com/muscle/) [43]. The OTU abundance information was normalized using a standard sequence number corresponding to the sample with the fewest sequences. Subsequent analyses of alpha and beta diversity were performed on these normalized output data.

Five indices, Chao1, Shannon, Simpson, ACE, and good-coverage, were calculated with QIIME (Version 1.9.1, http://qiime.org/index.html) [44] and displayed using R software (Version 2.15.3). Chao1 (http://www.mothur.org/wiki/Chao) and ACE (http://www.mothur.org/wiki/Ace) indices were selected to identify community richness. Shannon (http://www.mothur.org/wiki/Shannon) and Simpson (http://www.mothur.org/wiki/Simpson) indices were used to identify community diversity. Good’s coverage (http://www.mothur.org/wiki/Coverage) was used to characterize sequencing depth. Beta diversity analysis was used to evaluate differences in species diversity among samples. Beta diversity values (weighted and unweighted unifrac) were calculated using QIIME (Version 1.9.1). A non-metric multidimensional scaling (NMDS) analysis was conducted using the Vegan software package in R software (Version 2.15.3). The paired t-test was used for statistical comparisons between sample groups. Differences were considered significant at *p* < 0.05 and highly significant at *p* ≤ 0.01.

## Supplementary information

**Additional file 1: Table S1.** Endophytic fungi richness and diversity in different samples. **Fig. S1.** Relative abundance of the dominant (> 0.1%) fungal classification (phylum, order, family and genus) in leaf niches (upper leaf, middle leaf, lower leaf) and rhizosphere soil during different years.

## Data Availability

The datasets used and/or analysed during the current study are available from the corresponding author on reasonable request.

## Abbreviations

SCZ: Shu Cha Zao
DNA: Deoxyribonucleic acid
cDNA: Complementary deoxyribonucleic acid
PCR: Polymerase chain reaction
NMDS: Non-metric multidimensional scaling
ITS: Internal transcribed spacer
OUTs: Operational taxonomic units
CTAB: Cetyl Trimethyl Ammonium Bromide
*p*: Level of significance
R^2^: Determination coefficient
