# Supplementary material for: Temporal and spatial pattern of endophytic fungi diversity of Camellia sinensis (cv. Shu Cha Zao)
Source: BMC Microbiol. 2020 Aug 28;20:270. doi: 10.1186/s12866-020-01941-1 (PMC7455901; doi:10.1186/s12866-020-01941-1)
Supplement: Supplementary file 1 — Additional file 1: Table S1. Endophytic fungi richness and diversity in different samples. Fig. S1. Relative abundance of the dominant (> 0.1%) fungal classification (phylum, order, family and genus) in leaf niches (upper leaf, middle leaf, lower leaf) and rhizosphere soil during different years. [file 12866_2020_1941_MOESM1_ESM.docx]

Supplementary materials

Table S1 Endophytic fungi richness and diversity in different samples

| Sample name | Shannon | Simpson | Chao1 | ACE | Goods_coverage |
| --- | --- | --- | --- | --- | --- |
| YS1.1 | 5.318 | 0.928 | 461.459 | 473.779 | 0.996 |
| YS1.2 | 5.765 | 0.948 | 800.741 | 844.751 | 0.992 |
| YS1.3 | 6.273 | 0.964 | 578.581 | 572.775 | 0.996 |
| YS1.4 | 5.496 | 0.928 | 837.509 | 875.258 | 0.992 |
| YS1.5 | 6.858 | 0.978 | 742.25 | 711.474 | 0.995 |
| YS2.1 | 4.798 | 0.932 | 385.154 | 390.169 | 0.996 |
| YS2.2 | 4.385 | 0.864 | 499.958 | 493.77 | 0.995 |
| YS2.3 | 4.324 | 0.875 | 440.25 | 451.272 | 0.996 |
| YS2.4 | 3.622 | 0.789 | 474.791 | 501.635 | 0.995 |
| YS2.5 | 3.173 | 0.692 | 336.528 | 324.923 | 0.997 |
| YS3.1 | 4.767 | 0.911 | 423.95 | 426.768 | 0.996 |
| YS3.2 | 5.253 | 0.935 | 513.5 | 482.166 | 0.996 |
| YS3.3 | 4.149 | 0.853 | 390.894 | 387.345 | 0.997 |
| YS3.4 | 4.499 | 0.89 | 408.429 | 417.983 | 0.996 |
| YS3.5 | 4.415 | 0.82 | 337.419 | 342.294 | 0.997 |
| YS4.1 | 3.72 | 0.793 | 246.682 | 240.221 | 0.998 |
| YS4.2 | 3.851 | 0.815 | 283.526 | 292.145 | 0.998 |
| YS4.3 | 4.087 | 0.818 | 361.286 | 372.355 | 0.997 |
| YS4.4 | 3.653 | 0.754 | 351.512 | 358.265 | 0.997 |
| YS4.5 | 4.056 | 0.812 | 311.8 | 321.376 | 0.998 |
| ES1.1 | 4.496 | 0.873 | 718.88 | 781.901 | 0.993 |
| ES1.2 | 4.814 | 0.916 | 408.2 | 383.647 | 0.997 |
| ES1.3 | 5.593 | 0.933 | 724.795 | 729.78 | 0.994 |
| ES1.4 | 6.038 | 0.953 | 712.183 | 715.044 | 0.995 |
| ES1.5 | 6.026 | 0.96 | 690.888 | 694.574 | 0.995 |
| ES2.1 | 3.767 | 0.778 | 373.75 | 372.265 | 0.997 |
| ES2.2 | 4.737 | 0.912 | 312.396 | 324.736 | 0.997 |
| ES2.3 | 3.96 | 0.8 | 384.632 | 402.474 | 0.996 |
| ES2.4 | 4.494 | 0.904 | 357.023 | 379.689 | 0.996 |
| ES2.5 | 3.508 | 0.732 | 574.357 | 545.31 | 0.994 |
| ES3.1 | 4.517 | 0.917 | 346.043 | 353.171 | 0.997 |
| ES3.2 | 3.117 | 0.739 | 276.902 | 294.027 | 0.997 |
| ES3.3 | 4.073 | 0.879 | 305.82 | 318.81 | 0.997 |
| ES3.4 | 4.126 | 0.89 | 489.04 | 410.404 | 0.996 |
| ES3.5 | 4.367 | 0.904 | 417.148 | 437.518 | 0.996 |
| ES4.1 | 5.017 | 0.929 | 491.2 | 479.994 | 0.996 |
| ES4.2 | 4.69 | 0.913 | 424.818 | 435.23 | 0.996 |
| ES4.3 | 4.154 | 0.87 | 360.03 | 346.875 | 0.997 |
| ES4.4 | 4.461 | 0.911 | 538 | 509.505 | 0.995 |
| ES4.5 | 3.674 | 0.828 | 440 | 443.058 | 0.995 |
| LS1.1 | 6.884 | 0.979 | 875.782 | 891.776 | 0.993 |
| LS1.2 | 6.961 | 0.978 | 1247.784 | 1287.917 | 0.987 |
| LS1.3 | 6.218 | 0.968 | 742.398 | 743.126 | 0.994 |
| LS1.4 | 6.944 | 0.979 | 879.346 | 871.765 | 0.994 |
| LS1.5 | 6.84 | 0.978 | 864.761 | 871.48 | 0.993 |
| LS2.1 | 3.456 | 0.814 | 345.429 | 356.705 | 0.996 |
| LS2.2 | 3.661 | 0.849 | 312.24 | 288.788 | 0.997 |
| LS2.3 | 3.509 | 0.83 | 320.583 | 361.787 | 0.996 |
| LS2.4 | 3.293 | 0.778 | 302.717 | 313.944 | 0.997 |
| LS2.5 | 3.877 | 0.864 | 282.561 | 296.799 | 0.997 |
| LS3.1 | 2.961 | 0.779 | 195.371 | 223.738 | 0.998 |
| LS3.2 | 3.643 | 0.845 | 274.25 | 287.03 | 0.998 |
| LS3.3 | 3.692 | 0.842 | 389.438 | 423.897 | 0.996 |
| LS3.4 | 3.866 | 0.828 | 392.2 | 401.816 | 0.996 |
| LS3.5 | 3.289 | 0.794 | 323.516 | 328.504 | 0.997 |
| LS4.1 | 3.871 | 0.886 | 348.085 | 391.379 | 0.996 |
| LS4.2 | 3.016 | 0.786 | 207.75 | 207.118 | 0.998 |
| LS4.3 | 3.947 | 0.856 | 433.179 | 497.706 | 0.995 |
| LS4.4 | 4.072 | 0.876 | 420.443 | 452.957 | 0.996 |
| LS4.5 | 2.969 | 0.798 | 150.731 | 155.026 | 0.999 |

(YS, 1-year-old tea tree; ES, 20-year-old tea tree; LS, 60-year-old mother plant. 1, rhizosphere soil; 2, upper leaf; 3, middle leaf; 4, lower leaf.)


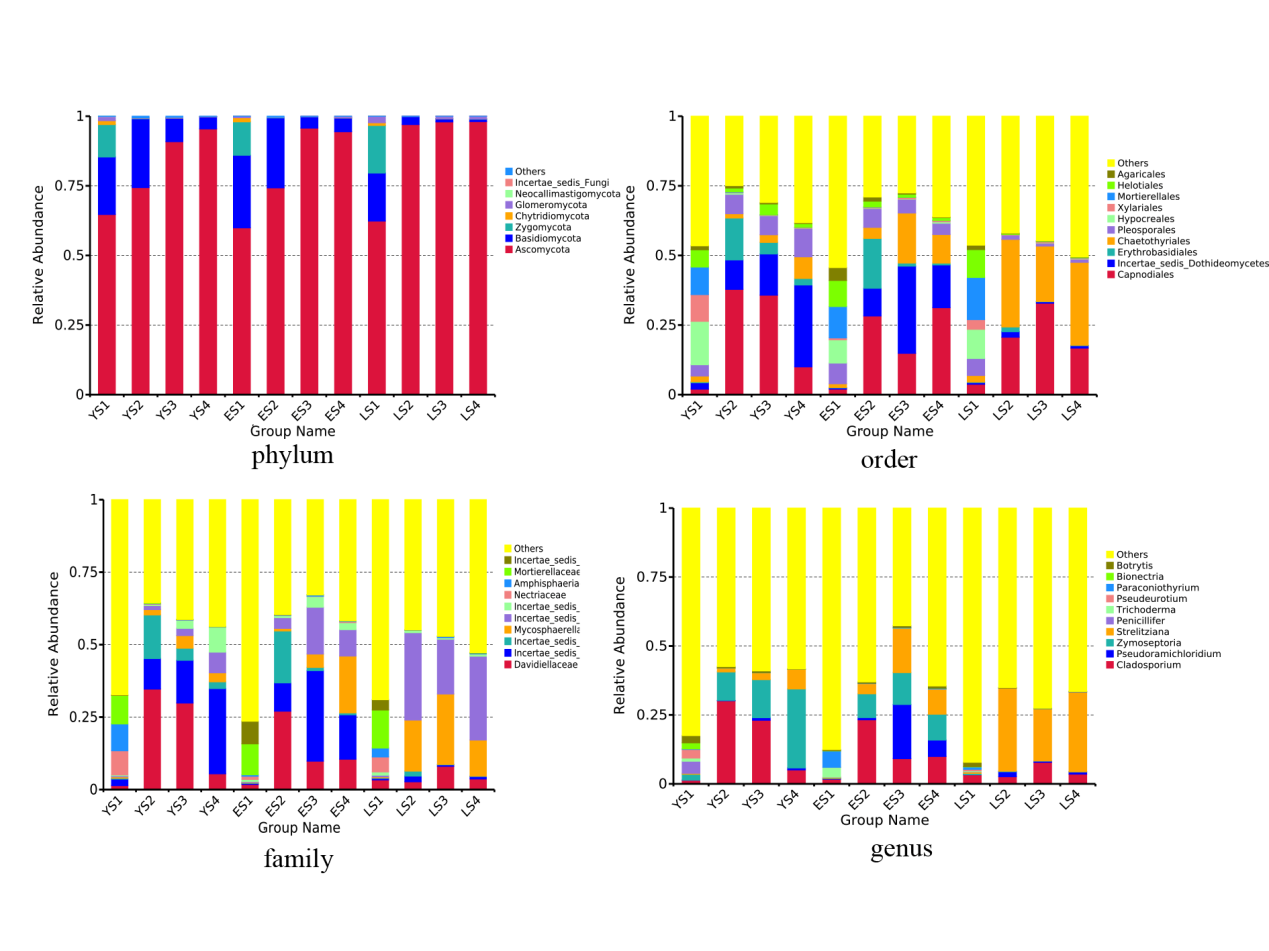


Fig. S1 Relative abundance of the dominant (>0.1%) fungal classification (phylum, order, family and genus) in leaf niches (upper leaf, middle leaf, lower leaf) and rhizosphere soil during different years.
